# Radiopacity Enhancements in Polymeric Implant Biomaterials: A Comprehensive Literature Review

**DOI:** 10.1021/acsbiomaterials.3c01667

**Published:** 2024-02-08

**Authors:** Crystal Kayaro Emonde, Max-Enno Eggers, Marcel Wichmann, Christof Hurschler, Max Ettinger, Berend Denkena

**Affiliations:** † Laboratory for Biomechanics and Biomaterials (LBB), Hannover Medical School, Anna-von-Borries-Strasse 1-7, 30625 Hannover, Germany; ‡ Institute of Production Engineering and Machine Tools, Leibniz University Hannover, An der Universität 2, 30823 Garbsen, Hannover, Germany; § Department of Orthopedic Surgery − DIAKOVERE Annastift, Hannover Medical School, Anna-von-Borries-Strasse 1-7, 30625 Hannover, Germany

**Keywords:** radiopacity, polymers, contrast agent, biocompatibility, radiolucent, implant

## Abstract

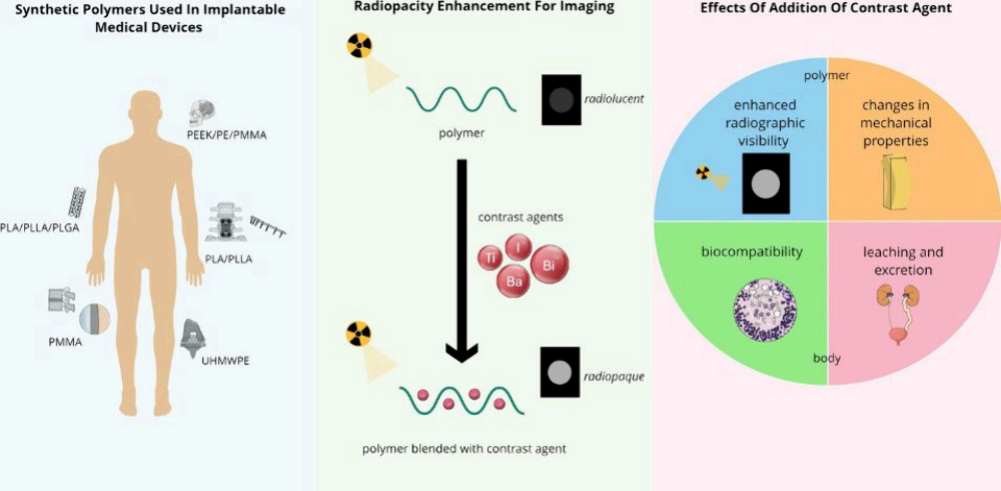

Polymers as biomaterials possess favorable properties, which include corrosion resistance, light weight, biocompatibility, ease of processing, low cost, and an ability to be easily tailored to meet specific applications. However, their inherent low X-ray attenuation, resulting from the low atomic numbers of their constituent elements, i.e., hydrogen (1), carbon (6), nitrogen (7), and oxygen (8), makes them difficult to visualize radiographically. Imparting radiopacity to radiolucent polymeric implants is necessary to enable noninvasive evaluation of implantable medical devices using conventional imaging methods. Numerous studies have undertaken this by blending various polymers with contrast agents consisting of heavy elements. The selection of an appropriate contrast agent is important, primarily to ensure that it does not cause detrimental effects to the relevant mechanical and physical properties of the polymer depending upon the intended application. Furthermore, its biocompatibility with adjacent tissues and its excretion from the body require thorough evaluation. We aimed to summarize the current knowledge on contrast agents incorporated into synthetic polymers in the context of implantable medical devices. While a single review was found that discussed radiopacity in polymeric biomaterials, the publication is outdated and does not address contemporary polymers employed in implant applications. Our review provides an up-to-date overview of contrast agents incorporated into synthetic medical polymers, encompassing both temporary and permanent implants. We expect that our results will significantly inform and guide the strategic selection of contrast agents, considering the specific requirements of implantable polymeric medical devices.

## Introduction

Synthetic polymers have extensive applications as biomaterials in medical implants. They can either be permanent, where their intended duration spans years, or temporary, where they are naturally biodegraded *in vivo* or removed upon healing.^1^ These polymers serve diverse functions, such as to restore the normal function of joints in arthroplasty, as drug delivery systems, or to provide physical and structural support to vascular systems.^2−5^ Polymers possess desirable characteristics such as biocompatibility, flexibility, corrosion resistance, ease of production, and various mechanical, physical, and chemical properties, which are considered beneficial depending on the intended application.^2^ Additionally, their properties can easily be modified to satisfy a wide range of requirements.^2,4−6^

Commonly used synthetic polymers in medical implants include polyethylene (PE), mainly comprised of ultra high molecular weight PE (UHMWPE), polyether ether ketone (PEEK), polytetrafluoroethylene (PTFE), poly(methyl methacrylate) (PMMA), polyurethane (PU), poly(lactic acid) (PLA), poly(l-lactic acid) (PLLA), poly(glycolic acid) (PGA), poly(lactic-co-glycolic acid) (PLGA), and polypropylene (PP).^4^ These polymers have found application in dental, orthopedic, vascular systems, and tissue engineering contexts.^2,5^

Routine monitoring of implants using conventional imaging techniques based on X-rays is a necessary approach to evaluate the performance and state of an implant postoperatively.^7−9^ Unlike metals and ceramics, which exhibit moderate-to-high contrast in radiographs relative to the surrounding tissues, most polymers are inherently radiolucent.^9−11^ Being radiolucent means that an object has low X-ray attenuation and will allow X-rays to pass through with little to no absorption, thereby limiting visibility. This radiolucency is dependent on a material’s atomic weight and electron density, which directly correlate to the level of X-ray attenuation.^11,12^ Polymers consist of repeating units of carbon, hydrogen, oxygen, and nitrogen atoms which have low atomic mass and electron density^12−15^ and, therefore, exhibit low attenuation of X-rays.

Numerous studies have investigated the incorporation of contrast agents to enhance the visibility of polymers in radiographs.^9,14,16^ Imparting radiopacity to polymers has proven to aid in monitoring the implant to allow precise surgical placement, evaluate biodegradation, or to detect malpositioning, migration, and wear.^17−20^ We aimed to conduct a thorough search of the literature to identify and summarize these contrast agents.

## Methods

The PubMed, ScienceDirect, and ResearchGate databases were searched from inception to current results for studies of polymeric biomaterials, including the terms “radiopaque polymers”, “contrast agents in”, “radiopacity in”, “X-ray contrast agent”, “contrast media”, and “radiopaque”, followed by the specific implantable device (e.g., stents, bone fixation devices, dental, bone cement) to limit the results to implantable devices.

## Results

### Quantifying Radiopacity

Heavy elements in the form of inorganic metal compounds, organic compounds, and pure metal powders are the most common contrast agents added to medical polymers. One major concern with these contrasts has been their detrimental effect on important mechanical and physical properties of the polymers.^14,16^ Another concern has been leaching out of the contrast agent, which could result in adverse reactions such as contrast-induced nephropathy or osteolysis, and should be kept below certain threshold levels.^16,20−23^

To quantify radiopacity, an aluminum 1,100 step wedge with uniform 1 mm thick steps graduated from 1 to 10 mm (many studies simplify the geometry of the wedge to reduce machining costs) is commonly used as the reference material.^24−27^ This wedge is placed beside the material of interest during X-ray image acquisition,^26^ and the grayscale values of the material of interest along with the step wedge are digitally analyzed. The radiopacity of the specimen is then referenced to the thickness of aluminum and expressed as the equivalent aluminum thickness (mmAl).^27−29^ The ASTM 5640-20 standard to test radiopacity^30^ recommends a minimum of 2 mmAl radiopacity for medical polymers for X-ray based techniques such as fluoroscopy, angiography, computed tomography (CT), and dual energy X-ray absorptiometry (DXA).^14,30,31^

**Figure 1:**
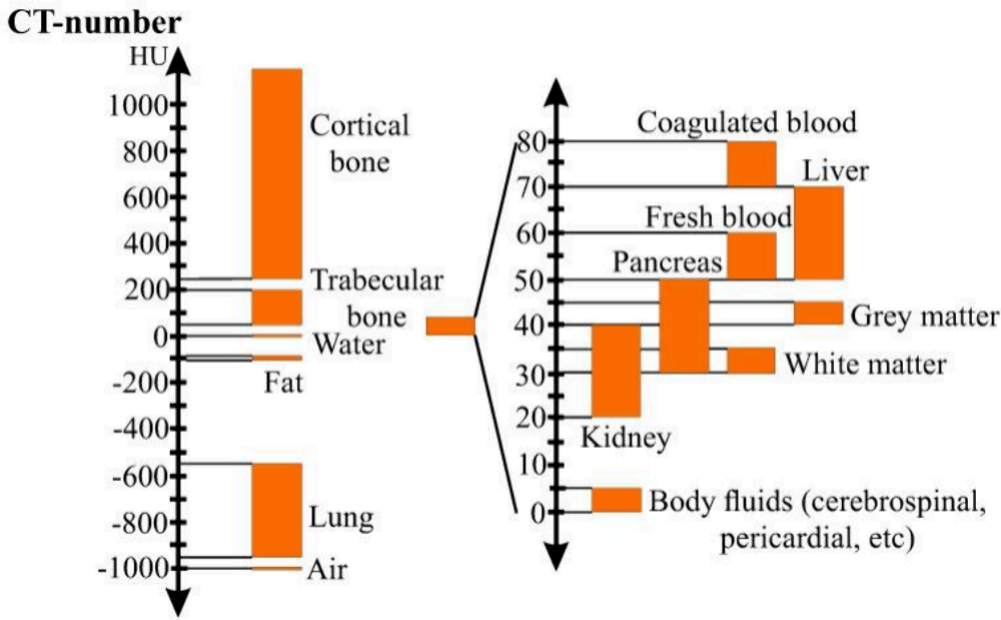
Hounsfield scale of different hard and soft tissues in the human body. Reproduced with permission from ref (34). Copyright 2020 MDPI.

Another common method of quantifying radiopacity is the Hounsfield Unit (HU), mainly used in CT (Figure 1).^32,33^ A material’s HU, the linear attenuation coefficients of distilled water and air, defined as 0 and −1,000 on the HU scale, respectively, together with the attenuation of the material (μ), is calculated according to the following equation:^32,33^
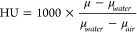
The higher the HU value, the higher the contrast of the material in a radiograph.

Furthermore, varying factors influence the choice of contrast agents in the medical field. The different categories of contrast agents require further investigation according to the anatomical region of application.

### Selection of Contrast Agent

**Figure 2:**
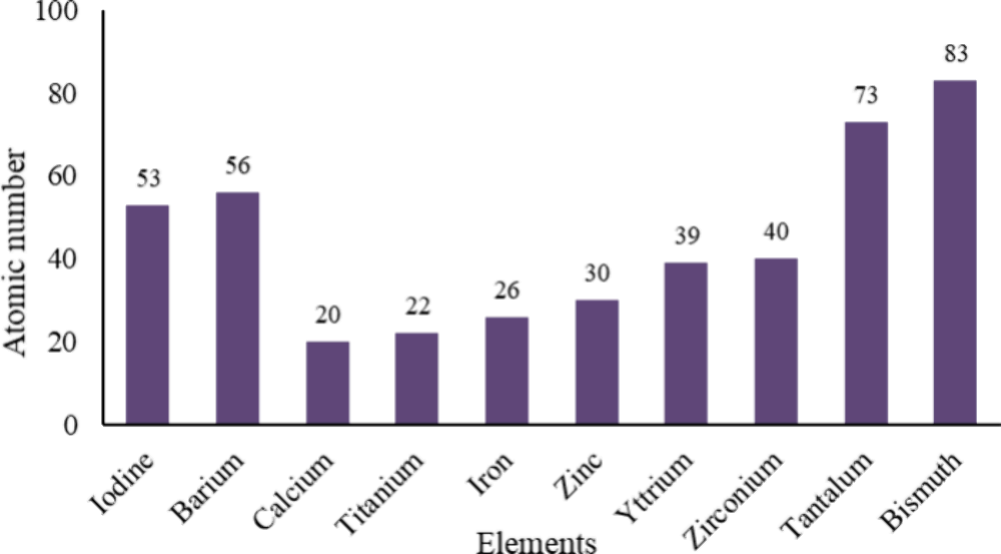
Atomic numbers of elements commonly contained in contrast agents.

Commonly used contrast agents in the medical field include compounds of iodine, barium, calcium, titanium, iron, zinc, yttrium, zirconium, tantalum, and bismuth *(*Figure 2), which are added to the polymer in specific quantities depending on the desired level of radiopacity.^9,14,35^ A higher atomic number, as well as a high contrast agent concentration, equates to a higher level of radiopacity.^36^ Some polymers require only moderate radioactivity to allow adequate monitoring of the implant without obstructing the underlying soft tissues. For instance, cranial implants should allow visualization of soft tissues in the CT “brain window”, which falls at 40 HU with a window width of 80.^18,37,38^ Others, such as vertebral bone cements and dental luting cements, require a much higher radiopacity which often exceeds the bony window level of 300 HU.^9,18,22,35^ The window width is the range of HU values which allows visualization of specific tissues, while the window level describes the midpoint of this range.^39^

The method of incorporation of contrast agents into the polymer matrix requires careful consideration. Radiopaque polymer composites can be fabricated in two ways, i.e., through physical blending methods such as injection molding, gel spinning, twin-screw extrusion, and solvent blending^11,18,22,26,40,41^ or through chemical synthesis, where the contrast is covalently bonded into the polymer structure.^7,14^ However, the use of chemical processes is complex and is considered impractical and uneconomical for medical implants.^7^

The level of radiopacity should be within the range of the surrounding anatomical structures, which includes both soft and hard tissues.^35^ Having insufficient or excessive radiopacity is often undesirable, as this could result in various complications such as misdiagnosis or obstruction of structures.^35,42^ Contrast agents that have been used clinically include inorganic compounds (primarily compounds of heavy metals), objects of pure metal, or iodine-containing compounds.^9,14^ Metal compounds negatively impact the physical-mechanical properties of polymers as they are only physically mixed in the matrix; thus, their distribution within the polymer is often inhomogeneous.^14,16,43,44^

It is important that the contrast agents are homogeneously distributed within the polymer matrix, to avoid the presence of agglomerated phases.^17^ Some studies suggest the use of nanosized particles that have been chemically functionalized to enable better integration of the two phases.^17,40,45−47^ Other studies prefer the use of iodinated nonionic compounds, which can be covalently bonded to the polymer and as a result deter the deterioration of the polymer’s properties and provide better stability of the contrast.^7,48^ Iodine-containing contrast agents normally consist of iodine molecules attached to an aromatic hydrocarbon group e.g., iodixanol (IDX), iohexol (IHX), iobitridol, and tri-iodobenzoic acid.^9,40,48,49^ When these iodine-containing hydrocarbons are attached to the backbone of the main polymer, the contrast agent becomes a part of the polymer.^14,16^ The advantage of this covalent bond is that a homogeneous and stable compound is formed and leaching can be minimized.^14^

The major limitation in the use of these iodine-containing contrast agents is their high cost, which would potentially reduce their application in industry.^50^ It is of great importance to tailor the concentration of the contrast agent in a way that will not compromise the desired mechanical and physical properties.

Contrast agents differ depending on the type of implant in which they are incorporated. As polymer-based bone cements are an integral part of implant surgery and are based on polymeric materials, they also require further discussion.

### Radiopacity in Polymer-Based Bone Cements

**Figure 3:**
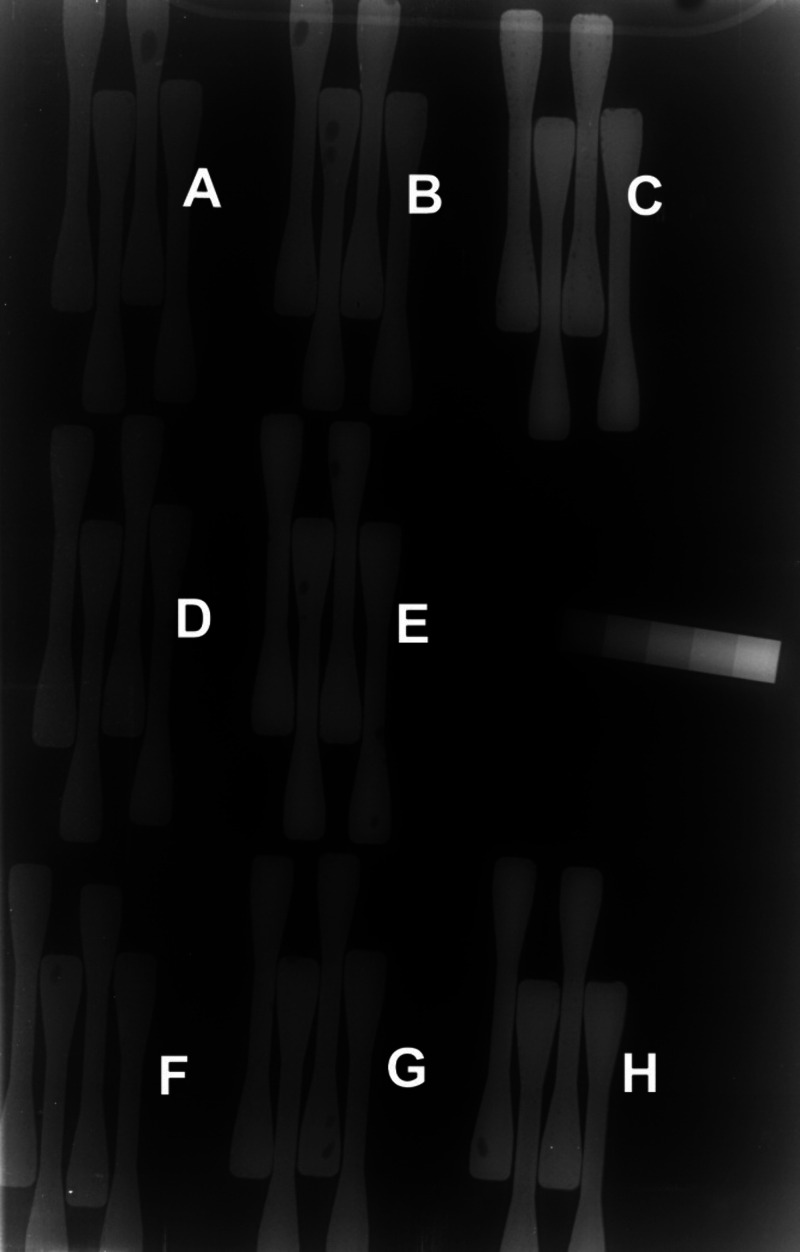
X-ray radiograph of specimens of commercial radiopaque bone cements, Palacos R (ZrO_2_), Simplex P (BaSO_4_), and nonpacified Palacos + IDX containing A, B, C = ZrO_2_ (5, 10, 15 wt %); D, E = BaSO_4_ (5, 10 wt %); F, G, H = IDX (5, 10, 15 wt %); and an aluminum wedge, all imaged with 0.1 m water phantom. Reproduced with permission from ref (12). Copyright 2004 John Wiley and Sons.

Radiopaque bone cements have been in use since the 1970s^12^ in joint replacement surgery and vertebroplasty and kyphoplasty, where they play the role of anchoring implants to the bone and relieving defects caused by vertebral fractures, respectively.^22,44^ Radiopaque bone cements are among the biomaterials in which contrast agents have been successfully incorporated to increase their radiographic visibility. Multiple bone cements exist commercially, which mostly contain inorganic heavy metal compounds, specifically BaSO_4_ and ZrO_2_ (Figure 3), as the contrast agents.^12,16,51^ Normally, these commercial bone cements have a contrast content ranging between 8–15 wt %.^12,52^ Vertebral and dental luting cements usually contain a higher contrast content in the order of 30 wt % or higher.^22^ Due to the comparatively lower viscosity/higher fluidity required in vertebroplasty, potential cement leakages present a life-threatening risk that warrants precise and accurate visualization of the cement *in vivo*.^23,51,53^

The addition of metal compounds has a detrimental effect on some of the mechanical properties of the cement.^14,16,54^ These include a reduction in the tensile strength and fracture toughness and fatigue life, which are relevant to the bone cement as it undergoes continuous loading.^16,43^ In the case of vertebral bone cements, which mainly differ from orthopedic bone cements in their much higher contrast content, the negative impact on the mechanical properties is elevated.^22^ The particles of ZrO_2_ are hard and abrasive and could be a source of third body wear, if they find their way to the articulating surfaces of knee and hip replacements.^12,51^ Another concern of metal compounds is the risk of leaching out of the polymer matrix over time, since they are only physically dispersed in the polymer. Leaching out of the contrast agent could trigger increased osteoclast activity and result in osteolysis and increased risk of early implant failure.^22,23,52^ The toxic nature of Ba^2+^ ions is also a source of concern;^44^ however, no recent studies report implant failures resulting from bone resorption due to BaSO_4_ leaching.

Contrast agents are also incorporated in bone cement spacers. Spacers are a temporary treatment option for periprosthetic infection in two stage revision arthroplasty procedures and are normally loaded with antibiotics.^55,56^ In the Copal Spacem (Heraeus Medical GmbH, Wehreim, Germany) articulating bone cement spacers, CaCO_3_ (15 wt %) is used as a contrast agent.^55^ Articulating spacers anticipate the release of cement particles during sliding; thus, CaCO_3_ is preferred over BaSO_4_ because it is nontoxic and less hazardous in the body than Ba^2+^ particles.^55,56^ Furthermore, as CaCO_3_ particles are soft and less abrasive, third body-induced wear is reduced. Müller et al. observed a 64% reduction in wear in a CaCO_3_-containing spacer compared to a BaSO_4_-containing spacer from the same manufacturer. It is worth noting, however, that CaCO_3_ exhibits lower radiopacity in comparison to BaSO_4_.^56^

Deb et al. and Hernandez et al. compared the potential benefits of using organic compounds of bismuth such as bismuth salicylate (BS) and triphenyl bismuth (TPB) as alternatives to BaSO_4_ in PMMA cements.^44,57^ These compounds exhibited better homogeneity and improved radiographic visibility, because of their solubility in the liquid phase (monomer) of the cement.^44,57^ Hernandez et al. specifically investigated the substitution of BaSO_4_ with BS for vertebroplasty cement and discovered that dissolving 10 wt % BS in the monomer of the radiolucent cement resulted in an enhanced cement with a lower setting temperature, better fluoroscopic visibility at the same concentration, and longer injection times, all desirable properties for vertebral cements. Additionally, the cement exhibited comparable biocompatibility to conventional cement.^57^ The addition of up to 10 wt % BS did not significantly alter the most relevant mechanical property for vertebral bone cement, i.e., compressive strength when compared to the commercial cement containing 10 wt % BaSO_4_. However, a significant reduction in the tensile strength with the addition of concentrations even as low as 5 wt % of BS was evident.^57^

Similarly, Deb et al. observed enhanced homogeneity and a lower polymerization temperature after dissolving TPB into the monomer of PMMA bone cement.^44^ The cement containing 10 wt % dissolved TPB exhibited superior mechanical properties (ultimate tensile strength, elastic modulus, and strain to failure) compared to the same cement containing a similar content of BaSO_4_. However, these properties reduced as the concentration of the contrast agent increased beyond this concentration. Dissolution of the contrast agent thus resulted in better mechanical properties of the cement due to better distribution of the contrast agent within the polymer matrix.^44^ Nevertheless, further investigation of the biocompatibility of TPB is recommended. Both studies found that dissolving the contrast agent in the monomer produced better homogeneity of the mixture, enhanced radiopacity, and enhanced mechanical properties compared to controls.^44,57^ Nevertheless, exceeding a 10 wt % contrast concentration had an adverse impact on the mechanical properties, attributed to a reduction in the solubility of the contrast agent, due to saturation of the monomer, rendering homogeneous mixing no longer feasible.^44^

The use of iodine-containing organic compounds as alternative contrast agents has also been explored in bone cements.^16,22,48^ Iodine-containing organic compounds have the advantage of being covalently bonded to the polymer matrix, resulting in better homogeneity and stability.^16,43,48^ Multiple studies have investigated 4-IEMA (4-iodobenzoyl-oxo-ethyl methacrylate), a crystalline iodine-containing monomer as an alternative radiopacifier in bone and vertebroplasty cement and found it to be a viable alternative.^16,22,48^ Le Ferrec et al. investigated iobitridol (Xenetix), a contrast agent normally injected into the body for radiographic imaging to enhance the fluoroscopic visibility of a calcium phosphate cement (CPC) for vertebroplasty.^49^ This water-soluble contrast was selected in place of BaSO_4_, to prevent the release of insoluble BaSO_4_ particles into the bloodstream during resorption of the CPC.^49^ Despite its nontoxicity, this contrast agent was rapidly released from the cement, making it unsuitable for long-term monitoring.^49^ Wang et al. compared the cellular response of two variants of water-soluble iodine contrast agents used in angiography by mixing PMMA + 10% IDX and PMMA + 10% IHX before polymerization and compared the formulations with conventional cements containing BaSO_4_ and ZrO_2_.^58^ The cements containing IDX and IHX were biocompatible in *in vitro* tests, with IHX exhibiting lower bone resorption compared to commercial cements. The limiting factor with these water-soluble contrast agents is the potential water uptake, which could cause the contrast agents to rapidly leach out and have a negative effect on the mechanical properties of PMMA.^14,41,59^

### Radiopacity in Joint Replacements

**Figure 4:**
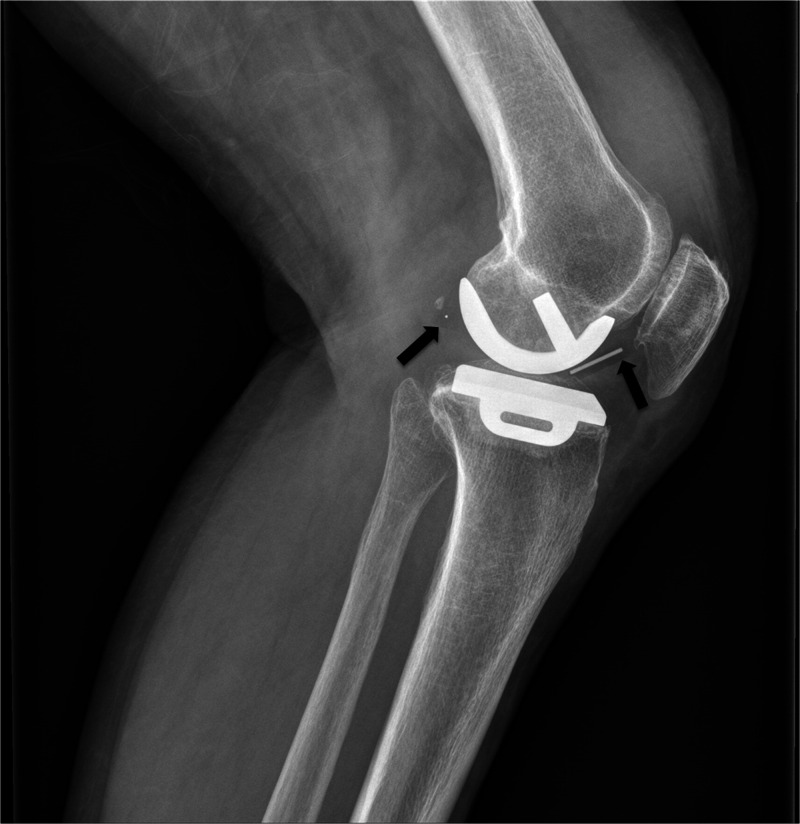
Anterior marker wire and posterior ball marker (shown with black arrows) enabled determination of fracture of this UHMWPE bearing. Reproduced with permission from ref (62). Copyright 2013 Elsevier.

The use of radiopaque markers in polymeric components of orthopedic implants, such as knee and hip replacements, has not been extensively investigated. Nevertheless, the Oxford Unicompartmental Knee Replacement (UKR) by Zimmer Biomet UHMWPE bearings is embedded with radiopaque markers in the form of metal wires made from titanium alloy, which are centrally positioned in the bearing. Another variant of this UKR employs a combination of the titanium alloy wire and tantalum marker balls placed anteriorly and posteriorly within the bearing.^60,61^ These radiopaque metal markers have proven important in relaying information regarding dislocation and fracture (Figure 4) of the UHMWPE components in radiographs, which would have otherwise gone undetected.^60,62−64^

However, the presence of these metallic rods is believed to have contributed to the fracture of the meniscal bearing.^62^ This was because the metal rods were inserted into slots created in the polymer, which caused localized reductions in thickness at these specific points and created areas of stress concentration.^60−62^ Another disadvantage of these markers is that they provided only partial visibility of the bearing.

Zaribaf et al. took a different approach, devising a method to enable radiographic visualization of the entire UHMWPE insert of a TKR using Lipiodol Ultra Fluid, an iodized oil used as a clinically injectable contrast agent.^14,65,66^ This was achieved by diffusing the oil into the polyethylene at an elevated temperature of 105 °C but below the melting point of the polymer (135 °C).^67^ This method had previously been used by Oral et al. to diffuse vitamin E oil into UHMWPE.^65,66,68,69^ Due to the load-bearing application of UHMWPE joint components, it was important that the mechanical properties of the insert remained unaltered by the diffusion of the oil. No significant changes in the mechanical and physical properties (i.e., tensile modulus, elongation at failure, ultimate tensile strength, crystallinity, and oxidative stability) were observed. Nonetheless, a minor alteration in the physical dimensions caused by swelling indicated that an extra machining phase would be necessary to achieve the desired insert geometry.^67^ Accelerated aging of the samples corresponding to 5 years *in vivo* revealed a reduction in the surface radiopacity of the samples from 1060 ± 53 HU to 600 ± 45 HU, which could compromise radiopacity of the insert relative to hard tissues.^18,70,71^ To mitigate leaching of the oil out of the polymer matrix, cross-linking of the polymer was suggested. The biocompatibility of the oil was not investigated, but was recommended for future studies. Nevertheless, existing studies reported that the iodine portion of Lipiodol is primarily excreted through the renal system, while the lipid component is excreted through the biliary system.^72^

### Radiopacity in Craniofacial Implants

Craniofacial implants aid in treating facial deformities caused by disease or trauma to the facial bones and tissues.^37,73^ Mild radiopacity is a requirement in various maxillofacial implants such as orbital reconstructions, where monitoring of the implant for malpositioning is crucial.^18,19^ Polyethylene is preferred over titanium in craniofacial implants due to its ease of shaping, biocompatibility, smoother edges, low cost, and lack of thermal sensitivity.^18,19,37,74,75^

Kozakiewicz et al. incorporated 2, 4, and 6% TiO_2_ in PE for lower orbital reconstruction to impart mild radiopacity relative to the surrounding fat and muscle tissues for X-ray CT.^18^ HU values of −83.2 ± 7.7 HU, – 25.2 ± 8.2 HU, and 67.9 ± 5.2 HU, respectively, were obtained, which fell within the range of fat and muscle (−70.1 ± 19.2 HU and 82.65 ± 7.1 HU, respectively). While a deterioration of the mechanical properties of PE was observed as a result of the addition of TiO_2_, i.e., reduced tensile and compressive strength, no cytotoxicity to human osteoblast cells was found, and the material was deemed suitable for application in craniomaxillofacial implants.^18^ Due to the low atomic number of Ti, TiO_2_ is only moderately radiopaque and a suitable contrast agent for applications where moderate radiopacity is required.^29^ Stryker has a commercially available product, MEDPOR Titan, a combination of high density polyethylene and titanium, which has proven to possess high flexibility, shape retention, strength, and radiographic visibility thanks to the incorporation of titanium.^74^

### Radiopacity in Bioresorbable Stents

The treatment of obstructed body vessels involves implanting a stent into the affected vessel to reopen the blocked pathway and restore its structure.^41,76^ To ensure proper positioning and detection of postoperative complications such as renarrowing of the vessel (restenosis), it is crucial for the stent to be visualized during and after implantation.^17,41,76^

Bioresorbable stents were developed as an alternative to metallic stents, which often exhibited problems such as restenosis, fractures, and a need for additional surgical removal procedures.^17,41,77^ Bioresorbable stents are typically made from synthetic biodegradable polymers, with PLLA being the most common choice due to its biodegradability and biocompatibility.^2,47^ Researchers initially incorporated radiopaque markers made of dense metals such as tantalum, gold, or platinum at the proximal and distal ends of stents to enable their visibility during medical imaging.^17,41,76,78^ However, these markers offered only partial visibility of the implant, which was insufficient in monitoring the stent *in vivo*. Moreover, there were concerns about metal pieces remaining in the body after resorption of the stent.^76^

BaSO_4_ is the preferred contrast agent, with concentrations typically ranging from 15% to 20% by weight or volume being incorporated into synthetic polymers such as PLGA and PLA.^17,47,79,80^ This contrast agent not only enhances radiopacity but has also been observed to dramatically enhance the mechanical properties of polymers, such as increasing the tensile and radial strength, as well as the modulus, making these polymeric stents mechanically comparable to metallic stents and enabling the user of thinner struts.^17,47^ However, some undesirable mechanical modifications have also resulted from the addition of BaSO_4_, which include reduced ductility and elongation at break.^17,47^ Therefore, it is essential to optimize the concentration of the contrast to achieve sufficient radiopacity without compromising stent functionality.^41,76^

A great concern for many researchers has been the elimination of BaSO_4_ particles from the body after the resorption of the stents.^47,73,79,81^ When administered orally as a contrast agent for radiographic procedures, BaSO_4_ only coats the gastrointestinal tract and can easily be excreted from the body.^7,82^ Outside the gastrointestinal tract, the toxicity of these particles is not fully known and remains under scrutiny, with various studies reporting and discouraging its use in the cardiovascular system.^7,47^ However, when evaluating its toxicity in the pancreas, Lämsä et al. likened the toxicity of 25 wt % BaSO_4_-laden PLA to that of steel, which is biologically inert in the human body.^79^

The use of iodine-containing organic compounds in stents has also been investigated.^7,41,76^ Wang et al. physically blended 40 wt % iohexol (IHX) and PLA and an additional small amount of poly(vinylpyrrolidone) (PVP), which served to facilitate the homogeneous mixing of the respective hydrophilic and hydrophobic phases.^7^ A high radiopacity of 4,680 HU was achieved, but a reduction in mechanical properties (tensile strength, modulus, and elongation at break) due to the effect of IHX was also observed, which PVP was found to regulate significantly.^7^ A high radiopacity is desirable to evaluate stent location and migration.^17^ Biocompatibility tests of radiopaque PLA in a rat model were found to be within the ISO 10993:2018 biocompatibility testing standards after 6 months.^7^

Ha et al. found no adverse reaction after 8 weeks of implantation of a polycaprolactone (PCL) stent containing 15% IHX in the iliac artery of a pig model.^41^ However, in both cases, a rapid release of the contrast agent was observed after incubating the iodine-containing stents in phosphate-buffered saline, which was accelerated by their solubility.^41,76^

The covalent bonding of iodine-containing contrast agents to the polymer backbone represents a viable strategy for long-term monitoring of biodegradable stents.^10,77^ By integrating the contrast agents into the polymer chain, visualization of the stent is made possible not only during placement but also throughout the entire degradation process.^77^

REVA Medical introduced a unique radiopaque bioresorbable drug-eluting coronary stent called Fantom made from TyroCore, a copolymer consisting of short-chain polylactic acid and tyrosine analogs with covalently bonded iodine.^83^ The stent offers the advantage of thinner struts, superior radial strength, and superior ductility compared to PLLA stents and radiopacity equivalent to commercially available cobalt–chromium metal stents.^84^ Clinical studies conducted at 6 and 12 months follow-up demonstrated favorable outcomes, with the stent exhibiting similar performance to contemporary metallic and PLLA counterparts.^83,84^ In addition, byproducts of the resorbed stent were reported to be safely excreted by the renal system.^77,84^

### Radiopacity in Implant Dentistry

Dental implants are generally made from metal, normally titanium (implant and abutment) and a ceramic or metallic crown, all of which possess adequate radiopacity for radiological imaging.^6,35,85,86^ For this reason, the use of contrast agents in oral implant dentistry mainly applies to filling and luting materials such as composite resins, endodontic sealers, and cements, which should be distinguishable from the surrounding anatomic structures.^25,42,87^ These materials require radiopacity for many reasons, which include evaluation of root canal fillings, recurrent caries, overhangs, voids, and remnant cement during cement removal.^27,35,42,88^

Filling and luting materials require differing levels of radiopacity depending on their surrounding anatomical structures.^35^ In dentistry, either transmission densitometers or digital image analyses are used to evaluate the optical density/radiopacity in dental radiographs.^27,89^ Dental (luting) cements are used for adhesive cementation, e.g., of crowns, abutments, veneers, and root posts,^42^ whereas filling materials are used to restore teeth.^36^ Insufficient or excessive radiopacity can lead to complications such as incorrect diagnostic assessment and obstruction of lesions.^35,36,90^ For root canal sealers, the American National Standards Institute/American Dental Association (ANSI/ADA57:2021) and ISO 6876:2012 recommend a minimum radiopacity equivalent to 2–3 mmAl, which is higher than that of dentin, which lies around 1 mmAl.^29,35,91^ On the other hand, ISO 4049 stipulates a minimum radiopacity of 1 mmAl for dental restorative resins, fillings, and luting materials.^36^ Metal compounds such as bismuth oxide, zinc oxide, barium sulfate, titanium oxide, tantalum oxide, calcium tungstate, and zirconium oxide are commonly used as radiopacifiers in root canal sealers.^35,87,91^ The choice of radiopacifiers for dental cements is important and should consider the cement base composition, which could comprise resin, glass ionomer, or polycarboxylate composites.^35^ The contrast agents typically used are similar to those used in sealers and include compounds of calcium, aluminum, zinc, strontium, yttrium, zirconium, barium, lanthanum, and ytterbium.^35,42^

Nevertheless, there is significant variation in the level of radiopacity of dental materials across different manufacturers.^25,36,89^ Some manufacturers only surpass the radiopacity of dentin (1 mmAl), while others marginally surpass that of enamel (2 mmAl) or by a factor of ≥3 mmAl.^27,36,42,87^

### Radiopacity in Spinal Implants

In spinal implants such as cages and rods, having a high level of radiopacity is not ideal, as it can lead to minor artifacts and hinder the accurate evaluation of bone growth during postoperative imaging.^92,93^ Two studies were found which explored radiopacity in spinal implant surgery, specifically concentrated on enhancing the radiopacity of UHMWPE sublaminar cables, which assist in guiding spinal growth during the treatment of early onset scoliosis (EOS).^26,94^ The use of metal sublaminar wires normally made from titanium poses the risk of breakages of the wire and metallosis and has been associated with neurological complications and artifacts during imaging.^26,94,95^ Bogie et al. blended 20 wt % bismuth trioxide (Bi_2_O_3_) into fibers of UHMWPE sublaminar cables and implanted the cables in sheep models for 24 weeks. Despite the cables sliding along the rods during bone growth, wear of the wire was minimal due to the low friction of the polymer.^92^ Histological studies revealed no adverse reactions, and there were no signs of wear particles from the wire, suggesting that no significant wear occurred within this time frame.^94,95^ The ultimate tensile strength and fatigue strength were found to be superior to clinically used sublaminar wires.^94^

**Figure 5:**
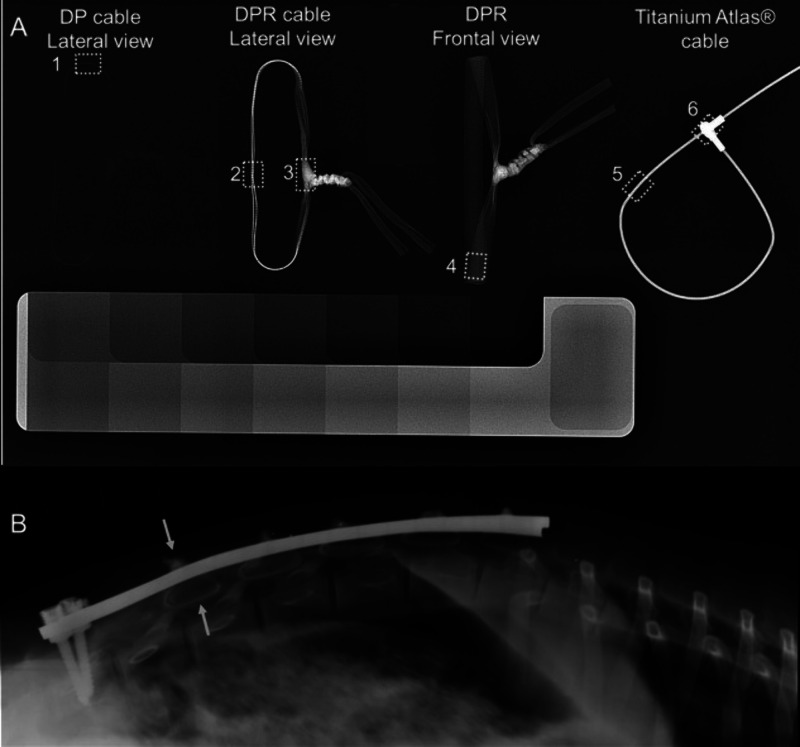
(A) Digital radiograph of radiolucent UHMWPE cable (1), UHMWPE cable incorporated with Bi_2_O_3_ particles in different views (2–4), and a titanium cable (5–6) relative to an aluminum step wedge (B) radiograph of the radiopaque UHMWPE cable implanted in a sheep spine. Reproduced with permission from ref (26). Copyright 2017 John Wiley and Sons.

In a study by Roth et al., the effects of physically incorporating Bi_2_O_3_ as a contrast agent were investigated (Figure 5). The mechanical properties (tensile strength and stiffness, fatigue strength, and creep elongation) of the same radiopaque UHMWPE wire were investigated,^26^ and the incorporation of bismuth trioxide did not significantly alter the mechanical properties of the wire when compared to the pure cable with no contrast. While bismuth compounds are nontoxic,^26^ the radiopaque cable exhibited substantially superior tensile and fatigue strength than the two commercially available cables used as controls.^26^ Furthermore, leaching studies conducted on sheep after 24 weeks of implantation showed that the amount of leached bismuth was well below the reported toxicity levels, with most of it being concentrated in the kidney, where bismuth(III) complexes are cleared by a protein with an affinity for bismuth.

### Radiopacity in Internal Fixation Systems

Two studies were found in which internal bone fixation devices were imparted with radiopacity. Choi et al. prepared 0.5 mm thick bioresorbable radiopaque composite layers of PLGA to BaSO_4_ compositions (1:10 and 1:3 w/w) and physically attached them on the surface of inion bone plates to allow radiographic visualization of the plates.^11^ This was to prevent the chemical alteration of the material of the bone plate. It was expected that the BaSO_4_ would be contained within the polymer and that the release of ions would be slowed down because of this, while both the plate and layer gradually degraded. Cytotoxicity studies on rabbits showed no difference in biocompatibility of bone plates containing both concentrations of layers in comparison to that of regular bone plates. Furthermore, both plates were visible for up to 8 weeks *in vivo*.^11^

**Figure 6:**
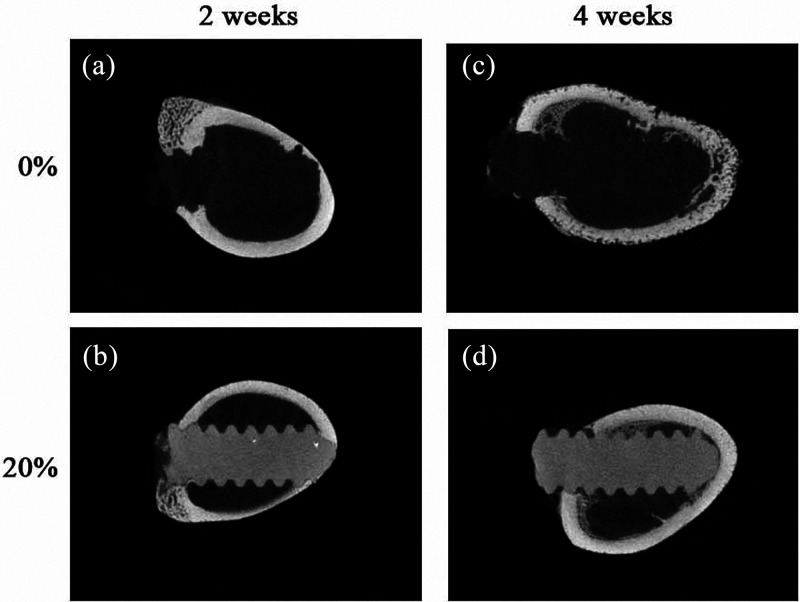
Micro-CT images of radiolucent PLLA bone screws (a, c) and radiopaque PLLA + 20 wt %. Fe_2_O_3_ particles (b, d) at 2 and 4 weeks, respectively, were implanted in white rabbits. Reproduced with permission from ref (40). Copyright 2015 W-J. Chang, Y.-H. Pan, J-J. Tzeng, T.-L. Wu, T.-H. Fong, S.-W. Feng, and H-M. Huang.

In another study, nanosized iron oxide (Fe_3_O_4_) particles were incorporated into PLLA by twin-screw extrusion and injection molding in concentrations of 0, 20, 30, 40 wt % to create radiopaque biodegradable bone screws (Figure 6).^40^ It was found that the 20 wt % Fe_3_O_4_ concentration was optimal for sufficient contrast without compromising the relevant mechanical properties of the polymer (flexural, ultimate tensile stress, and tensile strength), but higher concentrations reduced them significantly.^40^ Histology of the bone screws after implantation in white rabbits for 4 weeks revealed an osteogenic effect with 1.5% higher bone volume at the implant-bone interface, which could be attributed to the addition of Fe_3_O_4_.^40^

## Discussion

In clinical contexts, particularly in implantable medical devices, there is an increasing use of synthetic polymers due to their favorable characteristics, which include cost-effectiveness and their ability to be easily customized to achieve specific desired properties. Nevertheless, polymers lack radioactivity, an essential property that allows radiological monitoring of implants *in vivo*.

A comprehensive analysis of the existing literature was conducted to investigate current contrast agents used in polymeric implantable medical devices. A summary of the contrast agents highlighted in this review, their applications, and reported effects are summarized in Table 1. We found that two main categories of contrast agents were used to impart radiopacity in polymeric biomaterials: inorganic metal compounds and organic compounds, primarily those containing iodine.

**Table 1:** Summary of Contrast Agents Incorporated into Synthetic Polymers for Implantable Devices

*Contrast agent*	*Blending method*	*Polymer*	*Application*	*Content*	*Reported effects*	*Polymer biodegradable*	*Biological response*	*Ref*
*BaSO*_*4*_	Blended in powder phase	PMMA	Bone cement	9–15 wt %	Hard particles, third body wear, reduced tensile and flexural strength	No	Osteoclast formation	(12)
								(58)
								(52)
								(52)
	Blended in powder phase	PMMA	Vertebroplasty cement	30 wt %	Hard particles, third body wear, lower viscosity	No	Osteoclast formation	(8)
								(96)
								(56)
	Twin-screw microcompounding	PLLA	Bioresorbable stents	5–20 wt %	Increased tensile modulus and strength, decreased elongation at break and ductility	Yes	No adverse effects after 21 days	(45)
								(78)
	Magnetic stirring in organic solvent	PLGA	Bioresorbable stent	17.9 v/v %	Increased Young’s modulus, reduced elasticity, increased radial strength	Yes	Na	(17)
	Solution mixing	PLGA	Bone fixation plate	1:10 and 1:3 w/w PLGA:BaSO_4_	Radiopaque up to 56 days, BaSO_4_ leaching < 0.5 mg/day; insufficient to induce cytotoxicity	Yes	No adverse effects	(11)
*Lipiodol ultra fluid*	Immersion in oil at elevated temperature	UHMWPE	TKA insert	25 mL	Physical alteration–swelling, 54% reduction in surface radiopacity after 4 weeks	No	Na	(67)
*Iohexol(IHX)*	Stirring	PLA	Bioresorbable implants	40 wt %	Reduced tensile strength, elongation at break and increased tensile modulus, enhanced crystallinity, slower polymer degradation	Yes	Thin fiber capsule	(7)
	Blended in powder phase	PMMA	Bone cement	10 wt %	Better biocompatibility compared to conventional contrast agents	No	Osteoclast formation	(58)
*Iodixanol(IDX)*	Blended in powder phase	PMMA	Bone cement	10 wt %	Higher osteoclast formation than IHX	No	Osteoclast formation	(58)
*Iobitridol*	Dissolved in liquid phase	CPC	Bone cement	56 mg mL^–1^	Rapid release of contrast, no significant change in mechanical properties, no effect on injectability, cohesion or setting time	Yes	No adverse effects	(49)
*Iodinated diphenol*	Polymerization reaction	PLA diol	Coronary stent	<1% of 1 mL of iodine contrast	Increased ultimate tensile strength and elongation at break, long-term radiopacity	Yes	No adverse effects	(97)
*Bismuth salicylate(BS)*	Dissolved in liquid phase	PMMA	Vertebroplasty cement	10 w/w	Reduced compressive and tensile strength, reduced strain, lower setting temperature, increased radiopacity, longer injection time, Better compatibility than BaSO_4_	No	Na	(42)
								(55)
*Triphenyl bismuth(TPB)*	Dissolved in liquid phase	PMMA	Bone cement	10 wt %	Increased ultimate tensile strength, Young’s modulus and strain to failure, lower setting temperature, better homogeneity	No	Na	(44)
*Bismuth oxide Bi*_*2*_*O*_*3*_	Blended into fiber	UHMWPE	Sublaminar cables	20 wt %	Decreased tensile strength, limited leaching below toxic levels	No	No adverse effects	(26)
								(94)
*Titanium dioxide TiO*_*2*_	Blending	PE	Orbital implant	6%	Slight decrease in tensile strength and modulus, significant decrease in compressive strength and modulus, reduced hardness	No	No adverse effects	(18)
*Iron oxide Fe*_*3*_*O*_*4*_	Twin-screw extrusion	PLLA	Bone screws	20 wt %	Reduced flexural strength, increased crystallinity, increased thermal stability	Yes	Osteogenic effect, no adverse effects	(40)

Although physically blending these contrast agents into the polymer is the most prevalent and economical method to induce radiopacity, this approach has proven to be insufficient. The resultant mixtures often lack homogeneity, resulting in the aggregation of the different phases and thus compromising the radiopacity. As a result, it is necessary to incorporate a higher concentration of contrast agent than would otherwise be necessary. Some studies have suggested the use of biocompatible surface-modifying agents to mitigate this agglomeration and improve dispersion.^46^ The use of these surface modifiers has proven to allow for the use of lower concentrations of the contrast agent without compromising radiopacity.

An even higher radiopacity can be obtained from contrast agents that are soluble. This is possible if the contrast is soluble in a component of the polymer, such as the liquid phase in bone cement formulations. Dissolution provides better compatibility between the phases, resulting in a homogeneous distribution and allowing the use of an even lower concentration of contrast than surface modification for the same level of radiopacity.

Striking the right balance between the concentration of the contrast agent and the preservation of essential mechanical properties is crucial. Numerous studies have reported a change in mechanical properties such as Young’s modulus, tensile and compressive modulus and strength, hardness, and ductility (Table 1), specifically with increasing contrast agent concentrations. While these modifications are expected, it is necessary that the final values fall within the acceptable range of the respective implant’s standards or are comparable to what is currently commercially available. Reducing the amount of contrast agent to a concentration that would provide both acceptable radioactivity and mechanical properties is recommended.

The degree of radioactivity has been observed to directly correlate with the concentration and atomic number of the contrast agent. It is imperative that the desired radiopacity corresponds appropriately with the adjacent anatomical structures as different tissues within the human body require differing levels of radiopacity. Additionally, contrast toxicity, solubility, and excretion pathways must be considered. For instances where temporary radiopacity is required, water-soluble iodine contrast agents are advisible. This applies to implants, such as stents that are implanted within vascular systems. Clinically, these water-soluble iodine-containing contrast agents, such as iodixanol and iohexol, are administered intravenously and cleared by the renal system.

The cytotoxicity of the contrast needs to be extensively investigated and reported. In cases where permanent radiopacity is sought, securing the contrast agent in place through binders or cross-linking techniques should be considered. In such applications, the use of insoluble contrast agents, such as BaSO_4_, is recommended to prevent adverse biological reactions. However, the cytotoxicity of BaSO_4_ has not been characterized beyond the gastrointestinal tract.^7,47,79^ In situations where implants are subjected to mechanical articulation and wear particles should be avoided, the selection of a softer contrast agent may be advantageous.

To avoid adverse contrast-induced biological reactions, contrast concentrations must be maintained below the reported critical toxicological levels. Some studies propose polymer cross-linking to mitigate the leaching of contrast agents to tolerable levels, which would not only prevent adverse reactions but also increase the duration of radiopacity. Others propose covalent integration of the contrast agent into the polymer backbone, creating a stable molecular bond between the polymer and the contrast agent and enabling long-term radiopacity and reduced leaching. Additionally, certain contrast agents, such as Fe_2_O_3_, have exhibited unexpected therapeutic effects such as the stimulation of bone growth (osteogenesis). Exploring the use of such contrast agents and translating their benefits to other applications, such as in arthroplasty or bone cements, warrant further exploration.

In our review, we found that the use of polymeric biomaterials in implant devices is on the rise. Consequently, there has been increased interest in contrast agents that can be used to impart radiopacity to these polymers. The most common choice of contrast agent is well-established, clinically administered radiopaque agents such as BaSO_4_ and iodinated compounds. As these contrast agents have a long history of usage, their biocompatibility is sufficiently well-known and reported. Nevertheless, their incorporation in the polymer deteriorates mechanical properties, and their clearance from the body is still a matter of concern. In recent years, researchers have explored newer potential contrast agents, such as bismuth compounds, which are believed to possess better biocompatibility and provide increased radiopacity. The *in vivo* cytotoxicity of these contrast agents and their clearance from the body still require extensive investigation. Nevertheless, the findings of the studies within this review serve as a reference for future studies.

## Abbreviations

ADA: American Dental Association
ANSI: American National Standards Institute
ASTM: American Society for Testing and Materials
BS: bismuth salicylate
CPC: calcium phosphate cement
CT: computed tomography
DXA: dual energy X-ray absorptiometry
EOS: early onset scoliosis
HU: Hounsfield unit
IDX: iodixanol
IEMA: iodobenzoyl-oxo-ethyl methacrylate
IHX: iohexol
LBB: Laboratory for Biomechanics and Biomaterials
MISS: minimum invasive spine surgeries
PCL: polycaprolactone
PE: polyethylene
PEEK: polyether ether ketone
PGA: poly(glycolic acid)
PLA: poly(lactic acid)
PLLA: poly(l-lactic acid)
PLGA: poly(lactic-co-glycolic acid)
PMMA: poly(methyl methacrylate)
PP: polypropylene
PTFE: polytetrafluoroethylene
PU: polyurethane
PVP: poly(vinylpyrrolidone)
TPB: triphenyl bismuth
UHMWPE: ultrahigh molecular weight PE
UKR: unicompartmental knee replacement
